# The Library of Medicine

**Published:** 1840-07

**Authors:** 


### Art. XV.

*The Library of Medicine. Arranged and edited by* Alexander Tweedie, m.d., f.r.s. Vols. I-II. *Practical Medicine.—London,* 1840. 8vo, pp. 440, 353.

The plan of this work is taken from that of the Cabinet Cyclopædia of Dr. Lardner, and is, on the whole, a good one. Instead of the subjects being published in alphabetical order, as in most other encyclopædias and dictionaries, the different subjects which have more or less relation to one another, are arranged in groups and issued in separate volumes. Thus, in place of retracing his own footsteps in the Cyclopædia of Practical Medicine, or of following those of Dr. Copland in his Dictionary, by proceeding from a to b onwards, Dr. Tweedie collects into his first volume all the articles on *fever,* and into his second all those on *diseases of the nervous system.* He purposes to proceed in the same manner with the remaining articles, so as to collect, as far as it can be done, the allied subjects into volumes, and then group the volumes into series, according to the general nature of their contents, as of practical medicine, of physiology, &c. Dr. Tweedie's Library, like Dr. Lardner's Cyclopædia, further differs from the alphabetical collections in omitting entirely the lexicographical subjects and likewise a vast number of minor articles which are always contained in such works, and are scarcely to be found elsewhere. On this account, the range of information to be found in the work before us is much smaller than that in the Cyclopædia or Dictionary of Practical Medicine. Whether such a work as the present was wanted in the actual state of medical literature in this country, is a question which more concerns the publishers and proprietors than us.

We are of opinion that there can never be too many good books; and if the Library of Medicine comes under this category, it will not trouble us to know whether it may prove a successful or an unsuccessful mercantile speculation.

In any notice which we may take of the different volumes of this work as they appear, we shall, of course, hold the Editor no further responsible for the contents than as regards the selection of the writers and the arrangement of the subjects; but we have a small general quarrel with the work which we wish to settle *in limine*, and for the cause of which, we presume, the Editor is alone responsible. We refer to the paper and print—neither of which is what we think it should have been. The paper is not good enough, and the type is decidedly too small and the lines too crowded. Surely the learned Editor must have forgot that he, as well as ourselves, was past the years of aquiline or lyncean vision, else he never would have spread such amaurotic pages before his contemporaries. Youths of twenty may voyage through this perilous sea of ink in safety, but readers of double this age had better be content with gathering the pebbles on its shores. Or, had this muscæ-volitantes-type been inevitable, if our good Editor had condescended to break his lines in two, as the Cyclopædia did, as Hooper and Cooper did, as Copland did, as the Gazette and Lancet do, perhaps we might have been content, out of consideration for the student's pocket; but verily these broad, black pages are too much for our editorial eyes and patience.

In our next Number we hope to give a somewhat detailed account of the contents of these volumes. Our present engagements will only allow us to state generally that the articles—as far as our eyes have permitted us to examine them—are, on the whole, very good, and the Library of Medicine, consequently, a very good book, which we recommend to the attention of all our readers—under thirty. Before concluding, however, we will bestow a few words on the first two articles of the work, the Introduction by Dr. Symonds, and Inflammation by Dr. Alison, as they are, in some respects, complete in themselves, and bear only a general relation to the whole work.

These two articles comprise, we presume, all that this series is intended to contain of general pathology. Of the value of these essays in themselves we have a high opinion; but it is impossible to overlook their great disproportion to each other and to the rest of the work. We feel sure that to neither of these able writers is the fault to be attributed; but that it was inherent in the original plan.

The introduction is intended to "facilitate the study of diseases, by exhibiting to the reader a brief view of the more simple forms of morbid action, before leading him to the investigation of those complex phenomena which are the subjects of nosology." The particular objects dwelt upon by Dr. Symonds are: 1, the nature of disease in the abstract; 2, the leading principles which determine the association and succession of morbid phenomena; and 3, the elementary forms or *proximate principles* of disease. Now of all these, a summary (and, considering its narrow limits, a very full and comprehensive one,) is included within the space of fifty-one pages. Among the proximate principles, inflammation of course holds a distinguished place; and it is accordingly made the subject of a separate essay by Dr. Alison, which runs to the length of sixty-one pages of closer print than the introduction. Now we should scarcely wish to curtail this valuable chapter of a single sentence; but we feel it right to ask why other morbid actions of equal rank, and only inferior in importance on account of their less frequent occurrence, are passed over with the very brief notice, which is all that the limits we presume to have been imposed upon Dr. Symonds enabled him to bestow upon them? We shall look in vain, we fear, for any corresponding amplification of his brief but excellent account of tubercle and tuberculous cachexia, carcinoma and cancerous diathesis.

On the advantage of referring details on these subjects as much as possible to one general head, we need scarcely enlarge. In regard to inflammation, this has long been the practice of writers both on medicine and surgery; and since the equivalent value of other morbid alterations has been understood, the exclusive study of inflammation as the "one thing needful" has given place, in the most enlightened among our instructors, to a more comprehensive survey of the elementary forms of disease. Such will be found in Dr. Elliotson's lectures. We much regret, therefore, that in a work professing to be in all respects on a level with the time, so injurious an error should find countenance.

Of the details of these essays we can at present speak only in a very cursory manner. The introduction will be read by the intelligent student with great pleasure; as he will feel that he is both acquiring information from it and arranging by its means in a systematic form many ideas that were previously floating loosely in his mind. Its great condensation, however, appears to us rather opposed to the design which the author had in view; and we are doubtful of its fitness to communicate to the beginner that merely elementary knowledge which he requires. There seems to us an occasional *arrest of development* in some of the ideas, calculated to excite the thinking faculties of the intelligent reader, who can work them out for himself; but to perplex the younger student, for whom their connexion requires to be more fully displayed.

Disease is first viewed as consisting of morbid actions or phenomena occurring singly; and the relations of these to the healthy state are briefly considered. It is then regarded as consisting of groups of such actions or phenomena; and the chief circumstances to which these actions owe their association or connexion, are very ably, though briefly, treated of. We are inclined to think that it would avoid confusion if the term *disease* were restricted to the latter signification; and some other term applied to the several morbid phenomena which constitute it. We are much opposed, too, to the use of the term *proximate cause* to designate the essence of the disease; especially when immediately associated, as by Dr. Symonds, with *remote causes*, which mean something entirely different. At any rate, the very different acceptation of the word *cause* in the two instances should be thoroughly explained. We are disposed to think, moreover, that to limit the meaning of the word *pathology* to the consideration of the proximate causes of disease, or, in other words, of the primary morbid actions themselves, to the exclusion of the remote causes, and of the effects resulting from these primary actions, gives a very imperfect view of its extent. It is as if in physiology we were to consider the individual actions occurring in the several organs, without tracing their dependence upon external agents, or their connexion with each other. We have always regarded etiology and symptomatology essential parts of pathology; and as Dr. Symonds has referred (page 9) to physiology as an analogous instance, we are rather surprised that he should have overlooked this consideration. However, these are matters of comparatively trivial importance in comparison with the high practical value of the portion of the essay in which these terms occur; to these we have only thus referred, on account of the desire we feel that a settled and unambiguous meaning should be attached to all such forms of speech; and it is of little consequence in what sense an author uses them, provided he makes himself clearly understood.

The elementary forms of disease are arranged by Dr. Symonds according to their seats, or the parts in which they occur, into the following divisions: 1. Those of the *capillary vessels.* 2. Those of the *blood.* 3. Those of the extremities of the *nerves.* 4. Those of the *contractile fibres.* The diseases belonging to the first of these divisions are: *a*, alterations in the quality and the motion of the blood; *b*, diseased secretion; *c*, diseased nutrition. The second division includes excess or deficiency of blood throughout the system; and likewise abnormal states of that fluid. The last two divisions include "a host of morbid affections, which are often confounded with disorders of the circulation and other capillary actions, because they are often associated with them; but they may exist separately and imitate the others." They are disorders of feeling and of motion, and are often dependent upon diseases of the central organs of innervation; "but as they may exist separately, and be traced to a purely local origin, we have considered them deserving of a place among our pathological elements."

Now such a classification is doubtless useful, as all classifications except *very* artificial ones are capable of being made; but we must take leave to question its philosophical correctness. For example, we doubt if there is ever any purely local disorder of the nervous or muscular apparatus, which does not involve disordered nutrition. If we once admit the principle that all normal or physiological action is dependent upon two sets of conditions—an organized structure, possessed of certain properties which depend upon its peculiar form and character—and certain agents or stimuli external to it, by which these properties are called into operation, we do not see how the conclusion can be resisted, that the irregularity or morbid character of the action results from something out of order in the conditions. And, in the case of the muscular and nervous systems, if the disorder be not in the central organs, we do not see where else it can be than in the nutritive processes by which their properties are maintained. Again, changes in the nutritive and secreting processes are intimately connected (as we have formerly shown, Vol. VIII. p. 175, &c.) generally, indeed, in the relation of cause and effect, with changes in the quantity and motion of the blood through that individual part. Further, it is rather difficult to separate the processes of nutrition and secretion from each other, by any other line of demarcation than that the products of the former process immediately enter into organized structures, whilst those of the latter do not; yet in this classification, carcinoma is placed along with lymph, serum, and pus, as resulting from disorder of secretion. As all vital operations depend upon the continual reaction of the nutritive fluid and the solid parts which it permeates, so do all disorders originate (it appears to us) in a disturbance of the natural relation between these; and were we disposed to classify the primary forms of disease, we should distribute them under these two heads only. In seeking for the causes of this disturbance, we should not leave innervation out of view; but should regard it in the same light with other influences external to the part itself.

In offering these criticisms, we would again desire to express our high appreciation of the manner in which the several subjects are handled by Dr. Symonds. The outline he has given of the principal morbid conditions, arranged under the heads he has proposed, is executed with the hand of a master, who makes his sketches convey ideas as distinct and intelligible as can be acquired from the finished delineations of an inferior artist; and we venture to say that there are few of our readers who will not be benefited by the perusal of it.

On Dr. Alison's contribution we shall be very brief; since it appears to us so perfect and well-proportioned, that we find nothing in it to criticize. The speculative and the practical are blended together in that happy method which characterizes the best productions of this distinguished pathologist. In regard to the essential nature of inflammation, his views coincide very closely with those which we formerly expressed, and which were formed upon the principles laid down in his Outlines of Physiology and Pathology. He very properly devotes a little space at the outset to the proof that there is such a pathological condition, involving an alteration in the *vital* properties of the parts concerned, as that denominated inflammation; and to the refutation of the shallow dogmatisms of Magendie, and the more respectable but scarcely less erroneous views of Andral. We are sorry to perceive, however, that he makes no allusion to Dr. Macartney's opinions, which seem to us more worthy of consideration than these.

---

